# Comparison of multi-modal early oral nutrition for the tolerance of oral nutrition with conventional care after major abdominal surgery: a prospective, randomized, single-blind trial

**DOI:** 10.1186/s12937-017-0228-7

**Published:** 2017-02-10

**Authors:** Da-Li Sun, Wei-Ming Li, Shu-Min Li, Yun-Yun Cen, Qing-Wen Xu, Yi-Jun Li, Yan-Bo Sun, Yu-xing Qi, Yue-ying Lin, Ting Yang, Qi-Ping Lu, Peng-Yuan Xu

**Affiliations:** 1 Department of General Surgery, Wuhan Clinical School of Southern Medical University/Wuhan General Hospital of Guangzhou Military Command, Wuhan, 430070 China; 2 Department of Gastrointestinal Surgery, Second Affiliated Hospital of Kunming Medical University, Kunming, 650101 China; 3 Research Center for Surgical Clinical Nutrition in Yun-Nan Province, Kunming, 650101 China

**Keywords:** Multi-modal early oral nutrition (multimodal EON), Postoperative ileus (POI), Cost-effectiveness analysis

## Abstract

**Background & aims:**

Early oral nutrition (EON) has been shown to improve recovery of gastrointestinal function, length of stay and mortality after abdominal surgery; however, early oral nutrition often fails during the first week after surgery. Here, a multi-modal early oral nutrition program is introduced to promote recovery of gastrointestinal function and tolerance of oral nutrition.

**Methods:**

Consecutive patients scheduled for abdominal surgery were randomized to the multimodal EON group or a group receiving conventional care. The primary endpoint was the time of first defecation. The secondary endpoints were outcomes and the cost-effectiveness ratio in treating infectious complications. The rate of infectious-free patients was regarded as the index of effectiveness.

**Results:**

One hundred seven patients were randomly assigned to groups. Baseline characteristics were similar for both groups. In intention-to-treat analysis, the success rate of oral nutrition during the first week after surgery in the multimodal EON group was 44 (83.0%) versus 31 (57.4%) in the conventional care group (*P* = 0.004). Time to first defecation, time to flatus, recovery time of bowel sounds, and prolonged postoperative ileus were all less in the multimodal EON group (*P* < 0.05). The median postoperative length of stay in the multimodal EON group was 8 days (6, 12) versus 10 days (7, 18) in the conventional care group (*P* < 0.001). The total cost of treatment and nutritional support were also less in the multi-modal early oral nutrition group (*P* < 0.001). The effectiveness was 84.9 and 79.9% in the multimodal EON and conventional care group, respectively (*P* = 0.475). However, the cost-effectiveness ratio was USD 537.6 (506.1, 589.3) and USD 637.8 (593.9, 710.3), respectively (*P* < 0.001).

**Conclusion:**

The multi-modal early oral nutrition program was an effective way to improve tolerance of oral nutrition during the first week after surgery, decrease the length of stay and improve cost-effectiveness after abdominal surgery.

**Trial registration:**

Registration number: ChiCTR-TRC-14004395. Registered 15 March 2014.

## Introduction

An early start of oral nutrition is promoted in most patients undergoing abdominal surgery and is an core component that enhances recovery after surgery [1, 2]. An early start of oral nutrition within the first 24 h postoperatively is beneficial and has even been associated with a reduced mortality rate in comparison to no caloric intake [3]. However, the early start of oral nutrition is not successful *per se* in all patients after major abdominal surgery [2, 3] and is commonly delayed due to gastrointestinal dysfunction, including postoperative nausea, vomiting and bloating [3]. Most of these patients have to accept total parenteral nutrition to meet nutritional requirements because of intolerance of early oral nutrition during the first week after surgery [4]. Intolerance of early oral nutrition is also associated with prolonged hospital stays and increased costs [4, 5].

A multi-modal approach is introduced here to pave the way for early start of oral nutrition and improving gastrointestinal function. Through this concept, it is possible to improve the tolerance and dose of early oral nutrition without using feeding tubes. Therefore, we designed a prospective, randomized, single blind, controlled study to assess the impact of our multi-modal EON on gastrointestinal dysfunction, tolerance of early oral nutrition, clinical outcomes and cost-effectiveness ratios.

## Materials and methods

Between April 25, 2014 and April 1, 2016, 107 patients who were to undergo major abdominal surgery were enrolled in the clinical trial with a randomized, single-blind, controlled design. The protocol was approved by the ethics committee of the Second Affiliated Hospital of Kunming Medical University. The included patients were undergoing elective, radical oncologic surgery for gastric or colorectal cancer. The exclusion criteria were as follows: diabetes mellitus, severe pulmonary and cardiovascular disease and liver dysfunction, and Miles surgeries. Written informed consent was obtained before enrollment from each patient.

All patients had no pre-surgery medication or bowel preparation, and all patients fasted at least 8 h before surgery. Radical elective surgery was carried out by 3 senior surgeons. All of the patients received a standard anesthetic protocol and surgical management, prophylactic antibiotics, thoracic epidural for postoperative analgesia with patient controlled analgesia (fentanyl 50 μg/ml, 25 μg/ml per single dose in 6 min intervals) and restriction of intravenous fluid infusion. The nasogastric tube was inserted on the morning of surgery in all patients and was removed after the operation. No prokinetic pharmacotherapy was used in the 2 groups in the first 7 days postoperatively.

### Randomization

After enrollment, we assigned the patients into 4 groups with stratified random sampling: (1) a radical gastrectomy group, (2) a radical colectomy and radical resection for rectal cancer group, (3) a right hepatic resection group, and (4) a pancreaticoduodenectomy group. The patients in each subgroup were then randomly assigned to our multi-modal early oral nutrition group or to a conventional care group after the operation (Table 1). A randomization sequence list was provided by the Statistics Department of Kunming Medical University using SPSS 17.0 software (SPSS, Chicago, IL, USA). A researcher who was not involved with clinical care determined the treatment allocation by sequentially opening consecutively numbered, opaque, sealed envelopes.

**Table 1:** Differences between groups in baseline characteristics

	Multimodal EON group (*n* = 53)	Conventional care group (*n* = 54)	*P*
Sex, M:F	27:26	32:22	0.440
Age, years (mean ± SD)	56 ± 10	55 ± 10	0.624
BMI (mean ± SD)	22.2 ± 2.2	21.4 ± 2.3	0.069
Blood loss, ml (IQR)	300 (200, 450)	350 (250, 450)	0.231
Duration of operation, h (IQR)	3.5 (3, 4)	3.5 (3, 4)	0.211
Duration of ICU stay, h (IQR)	0 (0, 16)	0 (0, 17)	0.654
Duration of mechanical ventilation, h (IQR)	0 (0, 3)	0 (0, 5.3)	0.398
Duration of postoperative analgesia, h (IQR)	48 (41, 56)	48 (44, 55)	0.722
Types of operation performed			0.903
Radical gastrectomy, n	14	15	
Radical colectomy, n	11	12	
Radical resection for rectal cancer, n	8	11	
Right hepatic resection, n	13	11	
Pancreatoduodenectomy, n	7	5	
Tumor stage			0.442
T0-1, n	1	1	
T2, n	15	8	
T3, n	23	28	
T4, n	14	16	

### Interventions

The intervention was initiated on the first day after surgery and ended on the morning of the eighth day. The multimodal EON group received the following treatments (Table 1): (1) chewing sugar-free gum (30 min per session, 3 times per day) from the time the patients were returned to the ward and were awakened to the time of first defecation; (2) appetite stimulation (including playing a favourite food-related media program [30 min per session, 3 times per day], seeing the colours of and tasting favourite foods [5 min per session, at least 3–4 times per day], and watching other people dine [15 min per session, 3 times per day], among other stimuli) from the time of waking to the time of first defecation; (3) drinking water immediately on waking and drinking 100 ml juice (orange juice, apple juice or grape juice, containing 30 g of glucose) 6 h after surgery, oral administration of 300 ml enteral nutrition suspension (Peptisorb liquid, Nutricia) divided into 4–5 administrations initiated 12 h after surgery; the volume of the enteral nutrition suspension was increased to 500 ml at 24 h after surgery, and oral intake was gradually increased until normal requirements were reached. In the conventional care group, patients received the following treatments: they were sent to the ward postoperatively, intake of water and 300 ml enteral nutrition suspension (Peptisorb liquid, Nutricia) that was divided into 4–5 administrations was commenced after the first defecation, and oral intake was gradually increased until normal requirements were reached. Intake of water was performed after the operation according to the patients’ wishes.

Both regimens were isonitrogenous [0.2 g/kg (±0.01 Kcal) (±5%)] and isocaloric [24 Kcal/kg (±1.2 Kcal) (±5%)]. On the day of surgery, 6 h after the operation, parenteral nutrition was started in both groups. From day 2 postoperatively to day 7 postoperatively, if oral nutrition was not sufficient before 18 o’clock, then supplementation with parenteral nutrition was initiated after 18 o’clock. Vitamins and electrolytes were added as required. We recorded deviations to the protocol caused by a patient’s wishes, medical reasons or adverse events.

Blinded assessment of discharge criteria was performed in all of patients after postoperative day 2. The physicians assessing the outcomes (including 1 senior surgeon and 2 resident doctors) were blinded to the group assignments throughout the study. All patients were followed for 1 month after discharge by calling patients and their relatives or searching the medical records database of our hospital.

### The success rate of oral nutrition and recovery of gastrointestinal function

The primary endpoint was the success rate of oral nutrition, which was defined as the proportion of patients who tolerated oral nutrition supplying 80% full nutritional requirements and had no digestive symptoms (including vomiting, diarrhoea and abdominal distension) on day 7 postoperatively. First defecation, flatus, bowel sounds, bloating, vomiting, abdominal discomfort, postoperative ileus, time to tolerate ON supplying full nutritional requirements and reinsertion of the nasogastric tube also were recorded for the first 7 days postoperatively. The nasogastric tube was reinserted after two episodes of vomiting more than 100 ml over 24 h in the absence of bowel movements [6]. A postoperative ileus (POI) was defined as a transient cessation of coordinated bowel motility after surgical intervention that prevented effective transit of intestinal contents or tolerance of oral intake [7, 8]. POI was diagnosed when both criteria (passage of flatus or stool and tolerance of an oral diet) were not met before day 4 postoperatively [7]. A prolonged postoperative ileus was defined as a POI lasting more than 5 days for open surgery or more than 3 days for laparoscopic surgery [7, 8]. A recurrent postoperative ileus was defined as the occurrence of an ileus after an apparent resolution of the immediate postoperative POI [7, 8]. Intolerance of oral nutrition was defined as: (1) the presence of vomiting, diarrhoea, abdominal distension and/or an ileus after oral intake that led to halting oral intake [9]; and (2) oral nutrition supplying less than 80% of full nutritional requirements in 24 h [10].

### Complications

The most common complications after abdominal surgery were described, such as pneumonia, SIRS, septicaemia, intra-abdominal abscess, wound infection, urinary tract infection, central catheter infection, anastomotic blood, anastomotic leakage, wound dehiscence, intestinal obstruction, and deep venous thrombosis. An infectious complication was defined as the presence of recognized pathogens in typically sterile body tissues as confirmed by culture results and supported by clinical, radiologic, or hematologic evidence of infection [11].

### Length of stay (LOS)

Postoperative length of stay (PLOS) was defined as the duration between the date of operation and the date when discharge criteria were met. Discharge criteria included the ability to manage personal care and toilet activities, no fever, and no intravenous access [12]. When the patient’s condition met the above objective criteria, hospital stay was no longer considered to be sensitive to nutritional support, and the patient was considered to be discharged from a nutritional sense [12]. The actual length of stay (ALOS) was recorded.

### Cost

The cost of nutritional support was calculated as the total cost of all of the items related to nutritional support on the account statement, including nutritional products, disposal materials, consultation fee, infusion pumps, and catheters. The cost of infection-related complications (diagnosis and treatment by staff doctors who were unfamiliar with the protocol) was calculated as the total cost of all of the items related to the diagnosis and treatment of the infection on the account statement. The cost of the multimodal program was calculated as the total cost of all of the items (excluding nutritional support) related to the program, including fruit juice, fruits, the fee for preparation of individual multimedia materials (for playing food media programs), sugar-free gum, and other items. Indirect and intangible costs (pain and suffering) were not considered in this study. The total cost of treatment (C) = the cost of nutritional support and/or the cost of the multimodal program + the cost of infectious complications. Costs are expressed in USD (United States dollars). The cost-effectiveness analysis was performed from the payers’ perspective as described elsewhere [13].

### Sample size determination

Before the study was initiated, retrospective data (*n* = 20, unpublished results in patients with abdominal major surgeries in our hospital) indicated that the success rate of oral nutrition in the multi-modal EON care (*n* = 12) and conventional care (*n* = 8) were 9 (75.0%) and 4 (50.0%). We estimated that 100 patients would meet the inclusion criteria over the study period. If half of the patients were treated with the multi-modal EON, then according to a 2-sided alpha of 0.05, a sample size of 100 patients would provide more than 80% power to detect a relative reduction in the success rate of oral nutrition of 33.3% in the multi-modal early oral nutrition group compared to the conventional care group.

### Statistical analysis

The statistical analysis was performed using SPSS 17.0 (SPSS Inc., Chicago, USA) by a statistician who was also blinded to the group allocation in the Statistics Department of Kunming Medical University. After the data had been entered and verified, the statistician conducting the analyses was notified as to which group had received the multimodal program. Normally distributed data were expressed as mean ± SD and non-normally distributed variables were expressed as medians and interquartile ranges (IQRs). For continuous variables, the distribution of the data was analyzed for normality. A Student’s unpaired *t*-test was utilized for normally distributed numerical variables, and the Wilcoxon test was used for non-normally distributed numerical variables. Categorical data were analyzed using the chi-squared test or Fisher’s exact test. All of the statistical tests were two sided, and *P* < 0.05 was considered statistically significant.

## Results

### Patients and baseline characteristics

**Fig. 1:**
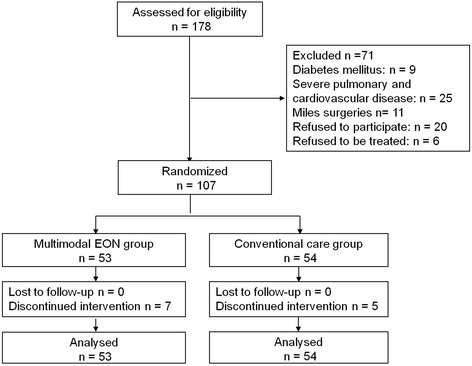
Allocation of experimental and control groups

**Fig. 2:**
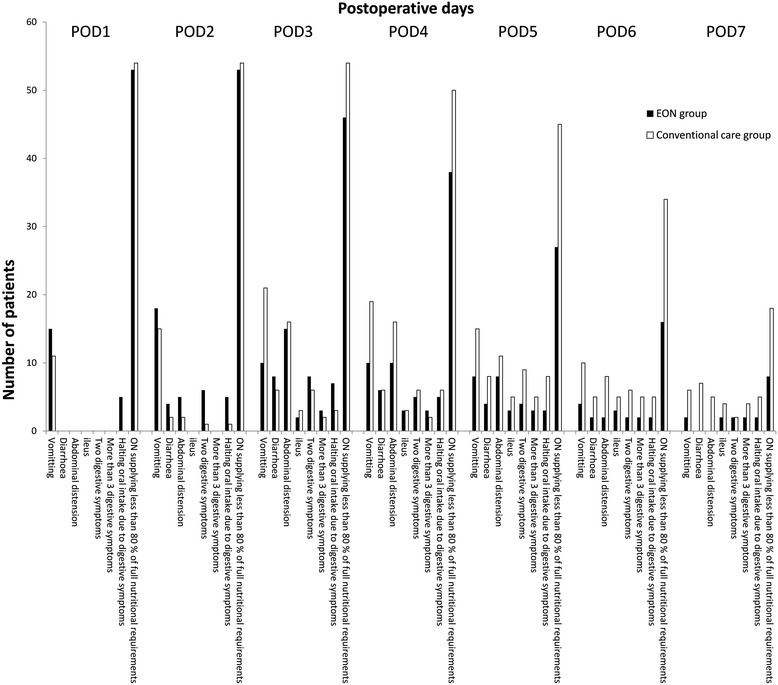
Intolerance of oral nutrition in the 2 groups

During the study period, there were 178 eligible patients. Seventy-one patients were excluded for various reasons, resulting in 107 patients being entered into the randomized program. Protocol deviations are shown in Figs. 1 and 2. Intervention was discontinued in 12 patients. Among the 12 patients, 2 patients in the multimodal EON group suffered obvious bloating and vomiting and anastomotic blood within 2 postoperative days; 5 patients in the multimodal EON group suffered prolonged postoperative ileus and anastomotic leakage after 3 postoperative days; 1 patient with anastomotic blood and 4 patients with anastomotic leakage in the conventional care group were changed to receive PN to supply nutritional requirements after 4 postoperative days. Eight patients (3 cases in the multimodal EON group and 5 in the conventional care group) were not contacted by telephone successfully. We searched the databases of our hospital and obtained their hospitalization data. Among 8 patients, 4 patients were treated with chemotherapy in the oncology department, one patient suffered ureteral calculi and received percutaneous nephrolithotomy in the department of urology, 1 patient suffered from femoral fractures after a traffic accident and was treated in the trauma department, and two patients were hospitalized in the cardiac department of internal medicine due to coronary heart disease. According to the medical records database of our hospital, no records of complications and readmission were associated with the abdominal surgeries and multimodal programs. Finally, no patient dropped out of the study or was lost to follow-up (Fig. 1). All analyses were performed according to the intention-to-treat principle.

The differences in baseline characteristics between the multimodal EON group and conventional care groups are shown in Table 1. No differences were found in demographic characteristics, operative procedures, or stage of the cancer between groups (*P* ≥ 0.05). The numbers of patients who underwent laparoscopic surgery in the multimodal EON group and the conventional care groups were 8 and 9, respectively (*P* = 0.824).

### The success rate of oral nutrition and recovery of gastrointestinal function

The success rate of oral nutrition in the multimodal EON group was 44 (83.0%) versus 31 (57.4%) in the conventional care group (*P* = 0.004; Table 2). According to the types of operation, 4 subgroups were analyzed, as shown in Table 2. The Bonferroni method was used to account for multiple comparisons among subgroups, but there was no significant difference between subgroups (*P* ≥ 0.05). In the conventional care group, we also did not find a significant difference between subgroups (*P* ≥ 0.05). Two subgroups (including laparoscopic surgery and open surgery) were analyzed, as shown in Table 2. In the multimodal EON group, the success rate of oral nutrition was 87.5% (*n* = 7) in the subgroup of laparoscopic surgery versus 82.2% (*n* = 37) in the open surgery subgroup (*P* = 1.000). In the conventional care group, the success rate of oral nutrition was 66.7% (*n* = 6) in subgroup of laparoscopic surgery versus 55.6% (*n* = 25) in the open surgery group (*P* = 0.717).

**Table 2:** Differences between groups in the success rates of oral nutrition

	Multimodal EON group (*n* = 53)	Conventional care group (*n* = 54)	*P* value
Types of operations			
Radical gastrectomy, n	10/14 (71.4%)	7/15 (46.7%)	0.176
Radical resection for colorectal cancer, n	18/19 (94.7%)	15/23 (65.2%)	0.027
Right hepatic resection, n	12/13 (92.3%)	7/11 (63.6%)	0.142
Pancreatoduodenectomy, n	4/7 (57.1%)	2/5 (40.0%)	1.000
Laparoscopic or open surgery			
Laparoscopic surgery	7/8 (87.5%)	6/9 (66.7%)	0.576
Open surgery	37/45 (82.2%)	25/45 (55.6%)	0.006
The total success rate of oral nutrition, n (%)	44/53 (83.0%)	31/44 (57.4%)	0.004

*EON* early oral nutrition

The rates of patients that halted oral intake due to digestive symptoms in postoperative days 3, 4, 5, 6 and 7 in the two groups were not significantly different (*P* = 0.202, *P* = 1.000, *P* = 0.119, *P* = 0.437 and *P* = 0.437, respectively). However, there were significant differences in the rates of patients with ON supplying less than 80% of full nutritional requirements in postoperative days 3, 4, 5, 6 and 7 in the two groups (*P* = 0.006, *P* = 0.005, *P* < 0.001, *P* = 0.001 and *P* = 0.028, respectively).

The difference in recovery of gastrointestinal function between the two groups is shown in Table 3. Time to first defecation and time to flatus occurred earlier in the multimodal EON group (*P* < 0.001) and bowel sounds returned sooner (*P* < 0.001). The number of patients with a prolonged postoperative ileus in the multimodal EON group was 5 (9.4%) versus 13 (24.1%) in the conventional care group (*P* = 0.043). There were no differences in the recurrent postoperative ileus and nasogastric tube reinsertion rates (*P* ≥ 0.05).

**Table 3:** Differences between groups in recovery of gastrointestinal function

Variables	Multimodal EON group (*n* = 53)	Conventional care group (*n* = 54)	*P* value
Time to first defecation, h (mean ± SD)	49 ± 7	62 ± 5	<0.001
Time to flatus, h (mean ± SD)	32.8 ± 5.3	42.9 ± 4.8	<0.001
Recovery time of bowel sounds, h (IQR)	24 (21, 27)	35 (31, 37)	<0.001
Prolonged postoperative ileus, n (%)	5 (9.4%)	13 (24.1%)	0.043
Recurrent postoperative ileus, n (%)	3 (5.7%)	6 (11.1%)	0.489
The nasogastric tube reinsertion rate, n (%)	4 (9.4%)	8 (14.8%)	0.359

EON early oral nutrition, IQR interquartile ranges

### Complications and length of stay

There was no decrease in the incidence of infectious complications in the multimodal EON group (8 of 53, 15%) compared with the conventional care group (11 of 54, 20%; *P* = 0.475). No significant differences were found between the two groups in the incidence of intraoperative complications and non-infectious complications (*P* ≥ 0.05; Table 4). There were a few minor intraoperative complications (including 2 cases of ecchymoma in the left arms in the multimodal EON group and 1 case of subcutaneous emphysema in the conventional care group) during the procedures. Only one death occurred (in the conventional care group) due to pneumonia and myocardial infarction. No patients who suffered any complications related to the last hospitalization in either group were readmitted within 30 days after discharge. The PLOS in the multimodal EON group was 8 (6, 12) days versus 10 (7, 18) days in the conventional care group (*P* < 0.001). There was no significant difference in ALOS between the multimodal EON group (26 [21.5–31.5] days) and the conventional care group (26 [26.0–29.3] days; *P* ≥ 0.05).

**Table 4:** Differences between groups in complications

	Multimodal EON group (*n* = 53)	Conventional care group (*n* = 54)	*P* value
No. of patients with infectious complications, n (%)	8 (15.1%)	11 (20.4%)	0.475
Pneumonia, n (%)	7 (13.2%)	9 (16.7%)	0.616
SIRS, n (%)	2 (3.8%)	9 (16.7%)	0.028
Septicaemia, n (%)	4 (7.5%)	3 (5.6%)	0.716
Intra-abdominal abscess, n (%)	2 (4.0%)	1 (1.9%)	1.000
Wound infection, n (%)	5 (9.4%)	4 (7.4%)	0.742
No. of patients with non-infectious complications, n (%)	10 (18.9%)	9 (16.7%)	0.766
Anastomotic blood, n (%)	2 (4.0%)	1 (1.9%)	1.000
Anastomotic leakage, n (%)	7 (13.2%)	6 (11.1%)	0.740
Wound dehiscence, n (%)	1 (1.9%)	1 (1.9%)	0.495
Intestinal obstruction, n (%)	0 (0.0%)	1 (1.9%)	1.000
Deep venous thrombosis, n (%)	2 (3.8%)	0 (0.0%)	0.243
No. of intraoperative complications, n (%)	2 (4.0%)	1 (1.9%)	1.000

EON early oral nutrition, SIRS indicates systemic inflammatory response syndrome

### Cost and cost/effectiveness

The differences between groups in cost and cost/effectiveness (C/E) in the treatment of infectious complications are shown in Table 5. Both the total cost of treatment/patient and the cost of nutritional support in the multimodal EON group were significantly less than in the conventional care group (10.4 and 9.4% less on average). For the multimodal EON group and the conventional care group, the rate of infectious complication-free patients was 84.9 and 79.9% (*P* = 0.475). The cost-effectiveness ratio was also less for the multimodal EON group than for the conventional care group (*P* < 0.001); therefore, the multimodal EON group was more cost effective than the conventional care.

**Table 5:** Differences between groups of cost and cost/effectiveness(C/E) in the treatment of infectious complications

	Multimodal EON group (*n* = 53)	Conventional care group (*n* = 54)	*P* value
Median cost of nutritional support/patient (IQR)	450.0 (426.7, 462.8)	496.8 (466.9, 526.4)	<0.001
Median cost of nutritional support/patient/day (IQR)	69.7 (61.8, 79.9)	67.2 (53.1, 78.6)	0.497
Median total cost of treatment/patient (C) (IQR)	456.4 (429.7, 500.3)	509.6 (474.5, 567.5)	<0.001
Effectiveness (E)	84.9%	79.9%	0.475
Cost/effectiveness (C/E) (IQR)	537.6 (506.1, 589.3)	637.8 (593.9, 710.3)	<0.001
Incremental cost-effectiveness ratio	1064		

EON early oral nutrition, IQR interquartile ranges, C cost, E effectiveness

To evaluate the reliability of the above findings, we conducted a sensitivity analysis by increasing the price of the preparations by 5% and observed that the results were consistent with the results provided in Table 5.

## Discussion

Multi-modal early oral nutrition increased the success rate of oral nutrition during the first week after surgery, and significantly decreased LOS and the cost of treatment. Thus, it was more cost-effective.

The success rate of oral nutrition was used to reflect tolerance of oral nutrition in our study, which included 2 aspects: (1) the gastrointestinal tract had the capacity to receive enough nutrition (at least 80% full nutritional requirements); and (2) the digestive ability of the gastrointestinal tract was also good, which did not cause digestive symptoms (including vomiting, diarrhoea and abdominal distension). The beneficial effects of multi-modal early oral nutrition on the success rate of oral nutrition and recovery of gastrointestinal function in this study were remarkable. Background mechanisms are thought to include the fact that several components of the multimodal protocol appeared to improve recovery of gastrointestinal function. Watching food-related media programs, perceiving the color and taste of favorite foods, watching other people dining, drinking small amounts of fruit juice, chewing gum, and the early provision of oral nutrition can stimulate the early brain phase and mouth and stomach phase, induce centrally mediated vagal effects [14], and promote the recovery from splanchnic nerve inhibition of motor activity due to surgery. In addition, early provision of oral nutrition may decrease inflammation and consequently decrease the duration of POI [15, 16]. In our study, SIRS in the first postoperative week was decreased significantly by multi-modal EON (Table 4). Chewing gum containing hexitol can produce an osmotic laxative effect [17, 18]. This hypothesis on background mechanisms was not investigated in this study.

In the subgroup analysis on the success rate of oral nutrition (Table 2), we analyzed the differences in the success rates of oral nutrition between different types of operations (including radical gastrectomy, radical resection for colorectal cancer, right hepatic resection and pancreatoduodenectomy). In the multimodal EON group, the success rates of oral nutrition in the subgroup of radical resection for colorectal cancer and the subgroup of right hepatic resection were more than 90%; but the success rates of oral nutrition were 71.4 and 57.1% in the radical gastrectomy subgroup and pancreatoduodenectomy subgroup. Even if we did not find a statistically significant difference, the differences in the success rates of oral nutrition in different types of operations were significant. Compared with resection for colorectal cancer and hepatic resection, reconstruction of the upper gastrointestinal tract after gastrectomy and pancreatoduodenectomy is more complicated, which also causes more disorder in the secretion of the digestive tract and more severe damage in nerves of the gastrointestinal tract. In theory, the tolerance of early oral nutrition in patients with upper digestive tract surgery was lower than that of the lower digestive tract.

Subgroup analysis on the success rate of oral nutrition in laparoscopic surgery and open surgery was also performed. In the multimodal EON group, the success rate of oral nutrition was 87.5% in the subgroup of laparoscopic surgery, which was slightly higher than that in the subgroup of open surgery (82.2%). This difference was not statistically significant. In previous studies, researchers found that laparoscopic surgery had an earlier return of bowel function when compared with open surgery [19–22]. The reduced use of opioid drugs during the operation, decreased intestinal manipulation and operation-related inflammation in laparoscopic surgery contributed to the result [21, 23, 24]. Whether it is able to increase the tolerance of early oral nutrition remains controversial. In a prospectively case-controlled study on effect of laparoscopic and open gastric bypass surgery on bowel function, the results showed the return of bowel movement and the time to first passage of gas in the laparoscopic group were shorter than that in the open surgery group (*P* < 0.05), but there was no significant difference in the time to oral food intake between the laparoscopic and open gastric bypass surgery groups (*P* = 0.06) [21]. In a meta-analysis on the clinical efficacy of laparoscopic and open surgery for rectal cancer [25], consuming liquid food data from 4 RCTs were pooled. The results indicated the time of consuming liquid food was 1.04 days earlier in the laparoscopic group than that in the open surgery group (*P* < 0.05). The inconsistent results from these studies, including our study, were related to the types of operations (the upper gastrointestinal tract or the lower digestive tract), the contents of early oral nutrition (normal food, fluid food, or enteral nutrition emulsion) and the different assessment methods for tolerance of oral nutrition.

However, it is prudent to qualify these results in subgroups because of inadequate sample sizes. In future research, expanding the sample sizes for different subgroups will further validate this result.

In our study, we found that multi-modal EON tended to reduce the risk of post infectious and non-infectious complications, but individual clinical complications failed to reach statistical significance, except for SIRS. These results are similar to the results of two recent meta-analyses of early enteral nutrition versus later commencement of feeding [26, 27]. In a meta-analysis [26], 14 RCTs represented 1,224 patients who underwent gastrointestinal surgery. The direction of the effect indicated that earlier feeding may reduce the risk of post-surgical complications without reaching statistical significance. Mortality was the only outcome showing a significant benefit, but was not necessarily associated with early commencement of feeding, as the reported cause of death was anastomotic leakage, reoperation, and acute myocardial infarction. In our study, one patient died due to pneumonia and myocardial infarction in the conventional care group, which was also not related to EON. In a meta-analysis including 7 RCTs with a total of 587 patients undergoing elective colorectal surgery [27], the researchers found that the risk of anastomotic dehiscence, pneumonia, wound infection, the rate of nasogastric tube reinsertion, vomiting, or mortality were not different between early enteral nutrition and later commencement of feeding. The total complications tended to be reduced EON (*P* = 0.04). Therefore, early enteral nutrition is safe in patients undergoing major abdominal surgeries, even if our protocol was moderately different from previous studies since it increased the multi-modal program to help the administration of EON.

In our study, PLOS under the multimodal protocol was on average 2 days less than that of patients managed with conventional care, which was consistent with the results of two meta-analyses [26, 27]. In previous studies, the PLOS was used as one of the evaluation indexes of early oral feeding [28–30], and PLOS was determined according to the discharge criteria [12, 28, 29]. Many factors might affect the length of stay. Some of these factors are altered by nutritional support, including mobilization and infectious complications, and some are not affected by nutritional support, including a planned admission for medical insurance policy reasons, delay until initiation of homecare or patient wishes [12] These factors may explain why there was no difference between the two groups regarding the ALOS.

Even if the incidence of infectious complications was not decreased by the multi-EON, it significantly improved the cost-effectiveness ratio in the treatment of infectious complications. Because the cost of oral nutrition was less than that of PN, the cost of nutritional support in the multimodal EON group was significantly decreased relative to that of the conventional care group (Table 5). The recovery of gastrointestinal function might also improve the treatment of infectious complications, thereby significantly decreasing the total cost of treatment per patient. Therefore, the multi-modal EON had a better therapeutic effect (Table 5). Compared with previous studies, the total cost of treatment was significantly low. The main reason was the total cost of treatment only included the cost of nutritional support (and/or the cost of the multimodal program) and the cost of infectious complications postoperatively in this study. This choice was made because infectious complications could be modified by nutritional support and non-infectious complications; e.g., deep venous thrombosis and anastomotic blood, were not sensitive to nutritional support. [12] Other hospitalization fees (related to operations, anesthesia, preoperative examinations and treatment, and the cost of non-infectious complications, etc.) were not calculated in the total cost of treatment. The secondary reason was that the incidence of infectious complications was 17.8% in all patients, which means that part of the cost of infectious complications in 82.2% of the patients was zero.

The present study had several methodologic differences from previous studies. *First,* in our multimodal protocol, we added new measures to stimulate appetite, such as watching favorite food-related media or other people dining and seeing, tasting favorite foods. *Second, w*e also used a single-blind design, but we blinded those who assessed outcomes and the statisticians. However, due to the characteristics of intervention programs, we could not blind the researchers and subjects. This strategy increased the power of the intervention. *Third,* we estimated the sample size before developing the study design and showed that it could effectively evaluate the effects of the multimodal protocol on the success rate of oral nutrition and recovery of gastrointestinal function. *Fourth,* we described the total range of costs in detail and used cost-effectiveness ratios to evaluate the pros and cons of the multimodal protocol for the success rate of oral nutrition and recovery of gastrointestinal function from a health economics perspective.

There were limitations in our study. First, even if some markers of recovery of gastrointestinal function (including time to first defecation, time to flatus and recovery time of bowel sounds) and the diagnosis of POI were commonly used in previous studies, these markers were somewhat subjective, which might cause inaccurate assessment of recovery regarding gastrointestinal function and incidence of POI. In a recent systematic review about the diagnosis of POI, the authors found that postoperative defecation together with tolerance of diet seemed to be the best clinical endpoint of POI and computed tomography had the best differential diagnostic value between POI and other complications [31]. Certainly, gastrointestinal motility monitoring and abdominal X-rays are also important affiliated means to diagnose POI. In future studies, relatively objective indicators, especially computed tomography, should be designed to accurately assess recovery of gastrointestinal function and POI. Second, we did not include a separate early oral nutrition group and a chewing gum group; therefore, we could not evaluate whether the multimodal protocol was more effective than early oral nutrition or chewing gum alone. In the multimodal protocol, we believe that each component may independently promote the recovery of gastrointestinal function via a different mechanism. Therefore, we suggest that any single component is unlikely to exceed the effect of the multimodal protocol on recovery of gastrointestinal function as an integrated process. Third, the cost was calculated based on a hospital computerized list for which the patients paid directly for the multimodal program and nutritional support, and the cost for treating infectious complications was also included. The other costs, such as the side effects of PN and/or EN, would probably affect the results, and the assessment of intangible costs (such as pain and suffering due to illness) would probably expand our findings. In addition, the economic parameters in our study may differ from country to country depending on the type of health care system and insurance reimbursement systems.

In this study, multi-modal EON was superior to conventional care in improving gastrointestinal function and tolerance of oral nutrition during the first week after surgery, decreasing the length of stay and improving cost-effectiveness. Therefore, we propose the use of multi-modal EON. We suggest a preoperative plan for multi-modal EON in patients with major abdominal surgery.

## Abbreviations

ALOS: Actual length of stay
C: Cost
C/E: Cost/Effectiveness
E: Effectiveness
EON: Early oral Nutrition
IQR: Interquartile range
ON: Oral nutrition
PLOS: Postoperative length of stay
PN: Parental Nutrition
PN: Parental nutrition
POI: Postoperative ileus
USD: United States Dollars

## References

[1] Kehlet H, Wilmore DW (2008). Evidence-based surgical care and the evolution of fast-track surgery. Ann Surg.

[2] Fearon KC, Ljungqvist O, Von Meyenfeldt M, Revhaug A, Dejong CH, Lassen K (2005). Enhanced recovery after surgery: a consensus review of clinical care for patients undergoing colonic resection. Clin Nutr.

[3] Lewis SJ, Andersen HK, Thomas S (2009). Early enteral nutrition within 24 h of intestinal surgery versus later commencement of feeding: a systematic review and meta-analysis. J Gastrointest Surg.

[4] Klaver YL, Nienhuijs SW, Nieuwenhuijzen GA, Rutten HJ, de Hingh IH (2008). Omentoplasty in rectal cancer surgery prolongs post-operative ileus. Int J Colorectal Dis.

[5] Blaser AR, Starkopf J, Kirsimägi Ü, Deane AM (2014). Definition, prevalence, and outcome of feeding intolerance in intensive care: a systematic review and meta-analysis. Acta Anaesthesiol Scand.

[6] Balayla J, Bujold E, Lapensée L, Mayrand MH, Sansregret A (2015). Early versus delayed postoperative feeding after major gynaecological surgery and its effects on clinical outcomes, patient satisfaction, and length of stay: a randomized controlled trial. J Obstet Gynaecol Can.

[7] Vather R, Trivedi S, Bissett I (2013). Defining postoperative ileus: results of a systematic review and global survey. J Gastrointest Surg.

[8] Bragg D, El-Sharkawy AM, Psaltis E, Maxwell-Armstrong CA, Lobo DN (2015). Postoperative ileus: recent developments in pathophysiology and management. Clin Nutr.

[9] Reignier J, Mercier E, Le Gouge A, Boulain T, Desachy A, Bellec F, Clavel M, Frat JP, Plantefeve G, Quenot JP, Lascarrou JB, Clinical Research in Intensive Care and Sepsis (CRICS) Group (2013). Effect of not monitoring residual gastric volume on risk of ventilator-associated pneumonia in adults receiving mechanical ventilation and early enteral feeding: a randomized controlled trial. JAMA.

[10] Woodcock NP, Zeigler D, Palmer MD, Buckley P, Mitchell CJ, MacFie J (2001). Enteral versus parenteral nutrition: a pragmatic study. Nutrition.

[11] American College of Chest Physicians/Society of Critical Care Medicine (1992). Consensus conference. Definitions for sepsis and organ failure and guidelines for the use of innovative therapies in sepsis. Crit Care Med.

[12] Johansen N, Kondrup J, Plum LM, Bak L, Nørregaard P, Bunch E (2004). Effect of nutritional support on clinical outcome in patients at nutritional risk. Clin Nutr.

[13] Braga M, Gianotti L (2005). Preoperative immunonutrition: cost-benefit analysis. J Parenter Enteral Nutr.

[14] Zaghiyan K, Felder S, Ovsepyan G, Murrell Z, Sokol T, Moore B (2013). A prospective randomized controlled trial of sugared chewing gum on gastrointestinal recovery after major colorectal surgery in patients managed with early enteral feeding. Dis Colon Rectum.

[15] Boelens PG, Heesakkers FF, Luyer MD, van Barneveld KW, de Hingh IH, Nieuwenhuijzen GA (2014). Reduction of postoperative ileus by early enteral nutrition in patients undergoing major rectal surgery: prospective, randomized, controlled trial. Ann Surg.

[16] Lubbers T, de Haan JJ, Luyer MD, Verbaeys I, Hadfoune M, Dejong CH (2010). Cholecystokinin/cholecystokinin-1 receptor-mediated peripheral activation of the afferent vagus by enteral nutrients attenuates inflammation in rats. Ann Surg.

[17] Tandeter H (2009). Hypothesis: hexitols in chewing gum may play a role in reducing postoperative ileus. Med Hypotheses.

[18] Bauditz J, Norman K, Biering H, Lochs H, Pirlich M (2008). Severe weight loss caused by chewing gum. BMJ.

[19] Schippers E, Ottinger AP, Anurov M, Polivoda M, Schumpelick V (1993). Laparoscopic cholecystectomy: a minor abdominal trauma?. World J Surg.

[20] Chen HH, Wexner SD, Iroatulam AJ, Pikarsky AJ, Alabaz O, Nogueras JJ, Nessim A, Weiss EG (2000). Laparoscopic colectomy compares favorably with colectomy by laparotomy for reduction of postoperative ileus. Dis Colon Rectum.

[21] El Shobary H, Christou N, Backman SB, Gvocdic B, Schricker T (2006). Effect of laparoscopic versus open gastric bypass surgery on postoperative pain and bowel function. Obes Surg.

[22] Morneau M, Boulanger J, Charlebois P, Latulippe JF, Lougnarath R, Thibault C, Gervais N (2013). Comité de l’Évolution des Pratiques en Oncologie. Laparoscopic versus open surgery for the treatment of colorectal cancer: a literature review and recommendations from the Comité de l’évolution des pratiques en oncologie. Can J Surg.

[23] Schwarz NT, Beer-Stolz D, Simmons RL, Bauer AJ (2002). Pathogenesis of paralytic ileus: intestinal manipulation opens a transient pathway between the intestinal lumen and the leukocytic infiltrate of the jejunal muscularis. Ann Surg.

[24] Liu SS, Carpenter RL, Mackey DC, Thirlby RC, Rupp SM, Shine TS, Feinglass NG, Metzger PP, Fulmer JT, Smith SL (1995). Effects of perioperative analgesic technique on rate of recovery after colon surgery. Anesthesiology.

[25] Zhao JK, Chen NZ, Zheng JB, He S, Sun XJ (2014). Laparoscopic versus open surgery for rectal cancer: results of a systematic review and meta-analysis on clinical efficacy. Mol Clin Oncol.

[26] Andersen HK, Lewis SJ, Thomas S (2006). Early enteral nutrition within 24 h of colorectal surgery versus later commencement of feeding for postoperative complications. Cochrane Database Syst Rev..

[27] Zhuang CL, Ye XZ, Zhang CJ, Dong QT, Chen BC, Yu Z (2013). Early versus traditional postoperative oral feeding in patients undergoing elective colorectal surgery: a meta-analysis of randomized clinical trials. Dig Surg.

[28] El Nakeeb A, Fikry A, El Metwally T, Fouda E, Youssef M, Ghazy H, Badr S, Khafagy W, Farid M (2009). Early oral feeding in patients undergoing elective colonic anastomosis. Int J Surg.

[29] da Fonseca LM, Profeta da Luz MM, Lacerda-Filho A, Correia MI, Gomes da Silva R (2011). A simplified rehabilitation program for patients undergoing elective colonic surgery – randomized controlled clinical trial. Int J Colorectal Dis.

[30] Dag A, Colak T, Turkmenoglu O, Gundogdu R, Aydin S (2011). A randomized controlled trial evaluating early versus traditional oral feeding after colorectal surgery. Clinics (Sao Paulo).

[31] Wu Z, Boersema GS, Dereci A, Menon AG, Jeekel J, Lange JF (2015). Clinical endpoint, early detection, and differential diagnosis of postoperative ileus: a systematic review of the literature. Eur Surg Res.

